# Supplementary material for: Molecular Russian dolls
Source: Nat Commun. 2018 Dec 10;9:5275. doi: 10.1038/s41467-018-07673-1 (PMC6288134; doi:10.1038/s41467-018-07673-1)
Supplement: Supplementary file 1 — Supplementary Information [file 41467_2018_7673_MOESM1_ESM.docx]

*Supplementary Information for*

**Molecular Russian Dolls**

Kang Cai^1^, Mark C. Lipke^1^, Zhichang Liu^2^, Jordan Nelson^1,3^, Tao Cheng^4^, Yi Shi^1^, Chuyang Cheng^1^, Dengke Shen^1^, Ji-Min Han^1,7^, Suneal Vemuri^1^, Yuanning Feng^1^, Charlotte L. Stern^1^, William A. Goddard III^3^, Michael R. Wasielewski^1,3^ and J. Fraser Stoddart^1,5,6*^

*^1^ Department of Chemistry, Northwestern University, 2145 Sheridan Road, Evanston, Illinois 60208, United States*

*^2^ School of Science, Westlake University, 18, Shilongshan Road, Hangzhou 310024, China*

*^3^Institute for Sustainability and Energy at Northwestern, Northwestern University, 2145 Sheridan Road, Evanston, Illinois 60208, United States*

*^4^ Materials and Process Simulation Center, California Institute of Technology (MC139-74), Pasadena, California 91125, United States*

*^5^ Institute for Molecular Design and Synthesis, Tianjin University, Nankai District, Tianjin 300092, P. R. China*

*^6^ School of Chemistry, University of New South Wales, Sydney, NSW 2052, Australia.*

^7^ Current address: Stake Key Laboratory of Explosion Science and Technology of China, Beijing Institute of Technology, 5 South Zhongguancun Street, Beijing,100081, China.

* Email: [stoddart@northwestern.edu](mailto:stoddart@northwestern.edu)

**Supplementary Methods**

**General Methods.** All reagents were purchased from commercial suppliers and employed without further purification. The tetracationic cyclobis(paraquat-*p*-phenylene) tetrakis(hexafluorophos-phate) (**CBPQT**•4PF_6_) was prepared according to literature procedures.^1^ Thin layer chromatography (TLC) was performed on silica gel 60 F254 (E. Merck). Solvents used in experiments involving radicals were degassed using the freeze-pump-thaw method. UV-Vis-NIR Spectra were recorded on a Varian 100-Bio UV-Vis spectrophotometer in MeCN at room temperature. Routine nuclear magnetic resonance (NMR) spectra and room temperature 2D NMR were recorded on Varian P-Inova 500 spectrometers, with working frequencies of 500 MHz for ^1^H, and 125 MHz for ^13^C nuclei, respectively. Variable temperature ^1^H NMR spectra and low temperature ^1^H-^1^H COSY NMR were recorded using an Agilent DD2 spectrometer with a 600 MHz working frequency for ^1^H nuclei. Chemical shifts are reported in ppm relative to the signals corresponding to the residual nondeuterated solvents (CD_2_HCN: δ =1.94 ppm). High resolution mass spectra were measured on a Finnigan LCQ iontrap mass spectrometer (HR-ESI). Cyclic voltammetry (CV) experiments were carried out at room temperature in Ar-purged MeCN solutions with a Gamry Multipurpose instrument (Reference 600) interfaced to a PC. CV Experiments were performed using a glassy carbon working electrode (0.071 cm^2^). The electrode surface was polished with 0.05 µm alumina-water slurry on a felt surface immediately before use. The counter electrode was a Pt coil and the reference electrode was Ag/AgCl electrode. The concentration of supporting electrolyte (tetrabutylammonium hexafluorophosphate) was 0.1 M.

**
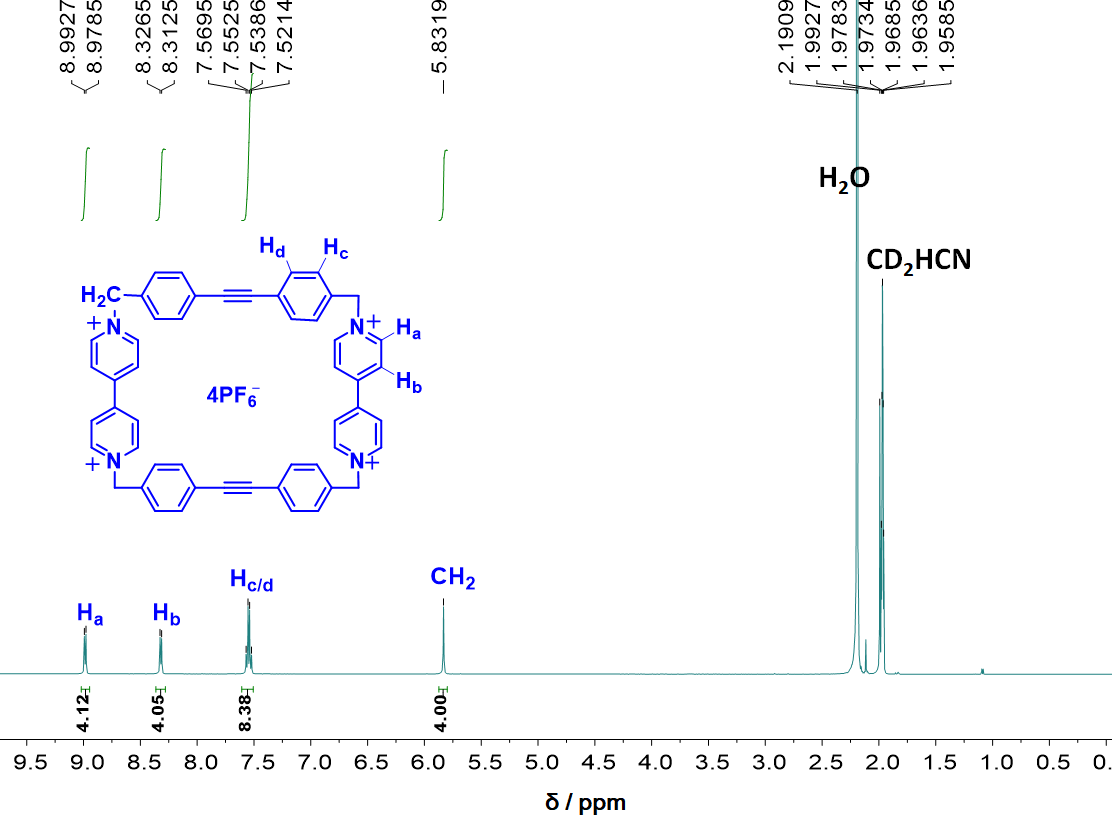
**

**Supplementary Figure 1.** ^1^H NMR Spectrum of **1**•4PF_6_ (500 MHz, CD_3_CN, 298 K)


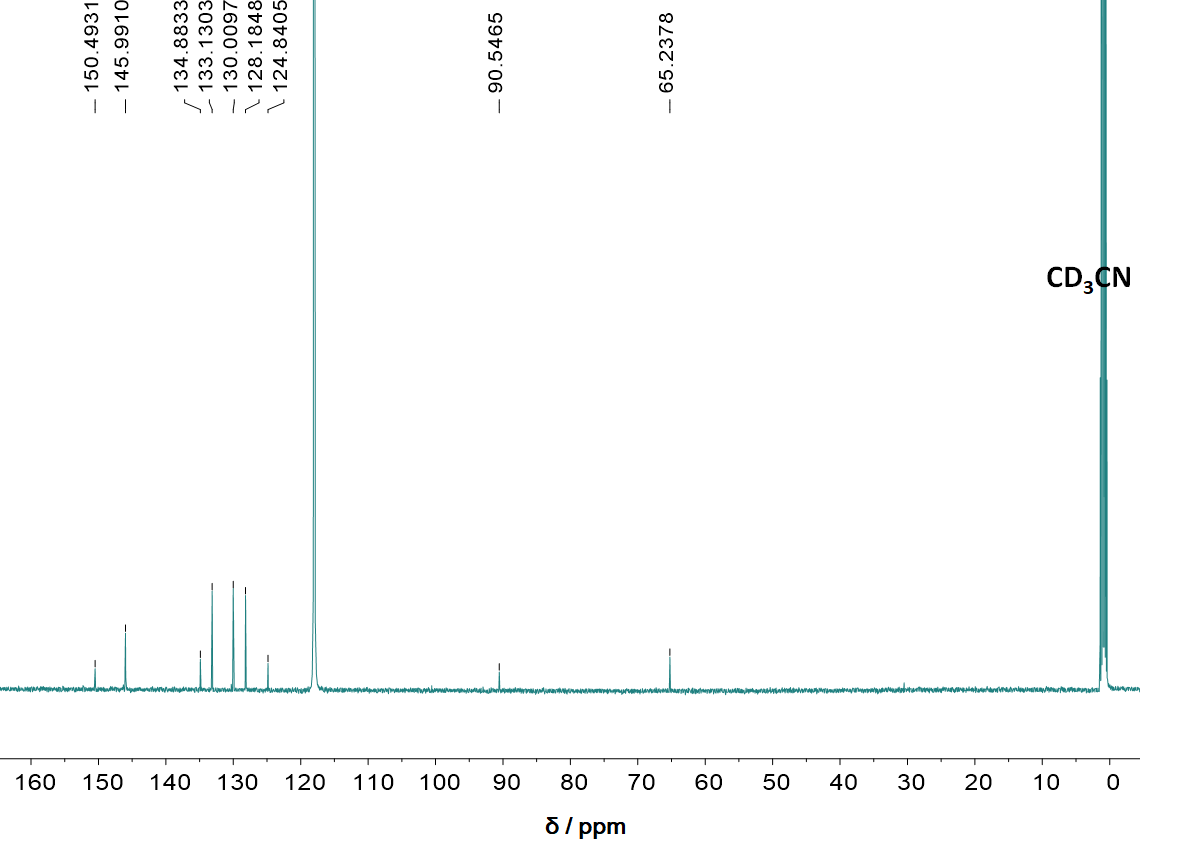


**Supplementary Figure 2.** ^13^C NMR Spectrum of **1**•4PF_6_ (125 MHz, CD_3_CN, 298 K)

**
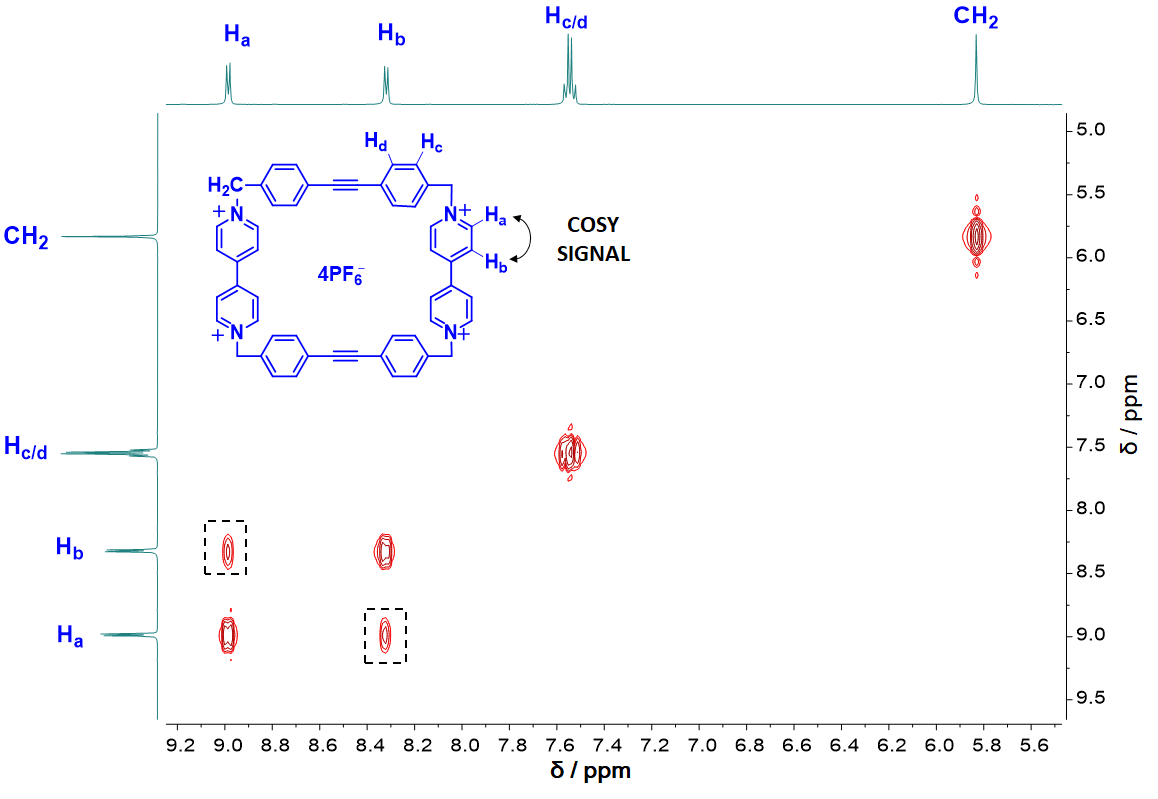
**

**Supplementary Figure 3.** ^1^H-^1^H COSY NMR Spectrum of **1**•4PF_6_ (500 MHz, CD_3_CN, 298 K)

**
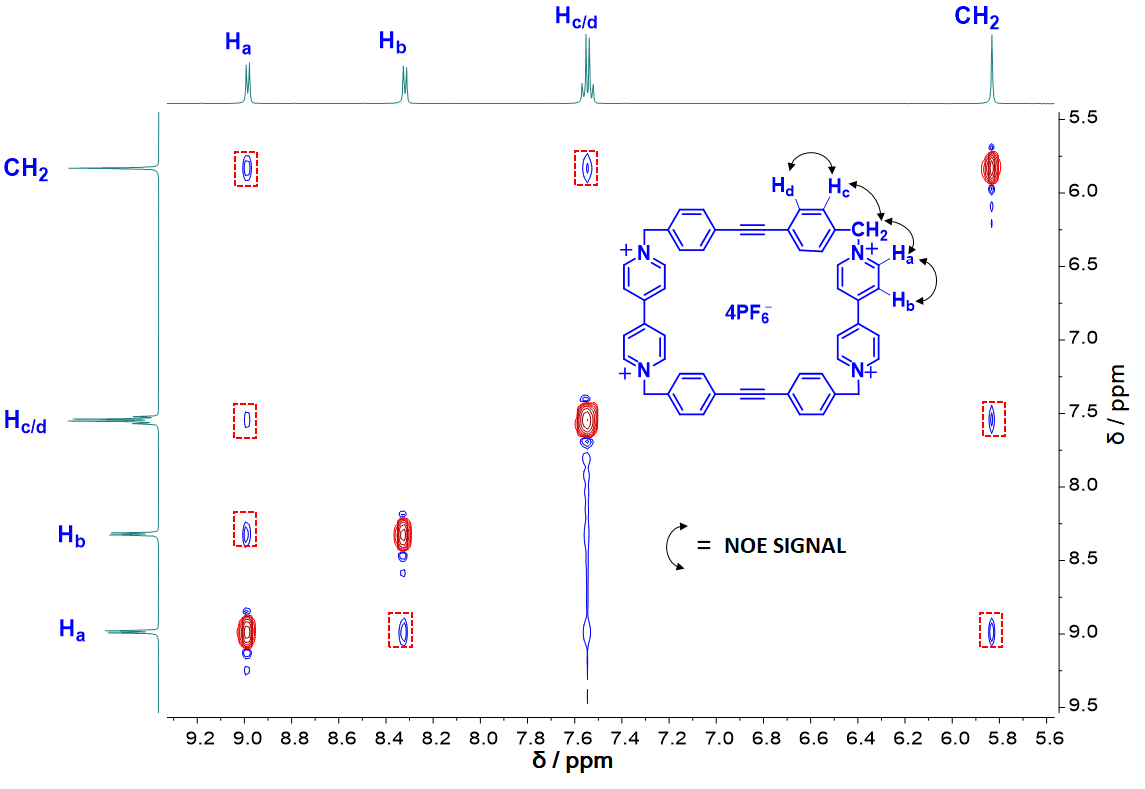
**

**Supplementary Figure 4.** ^1^H-^1^H NOESY NMR Spectrum of **1**•4PF_6_ (500 MHz, CD_3_CN, 298 K)


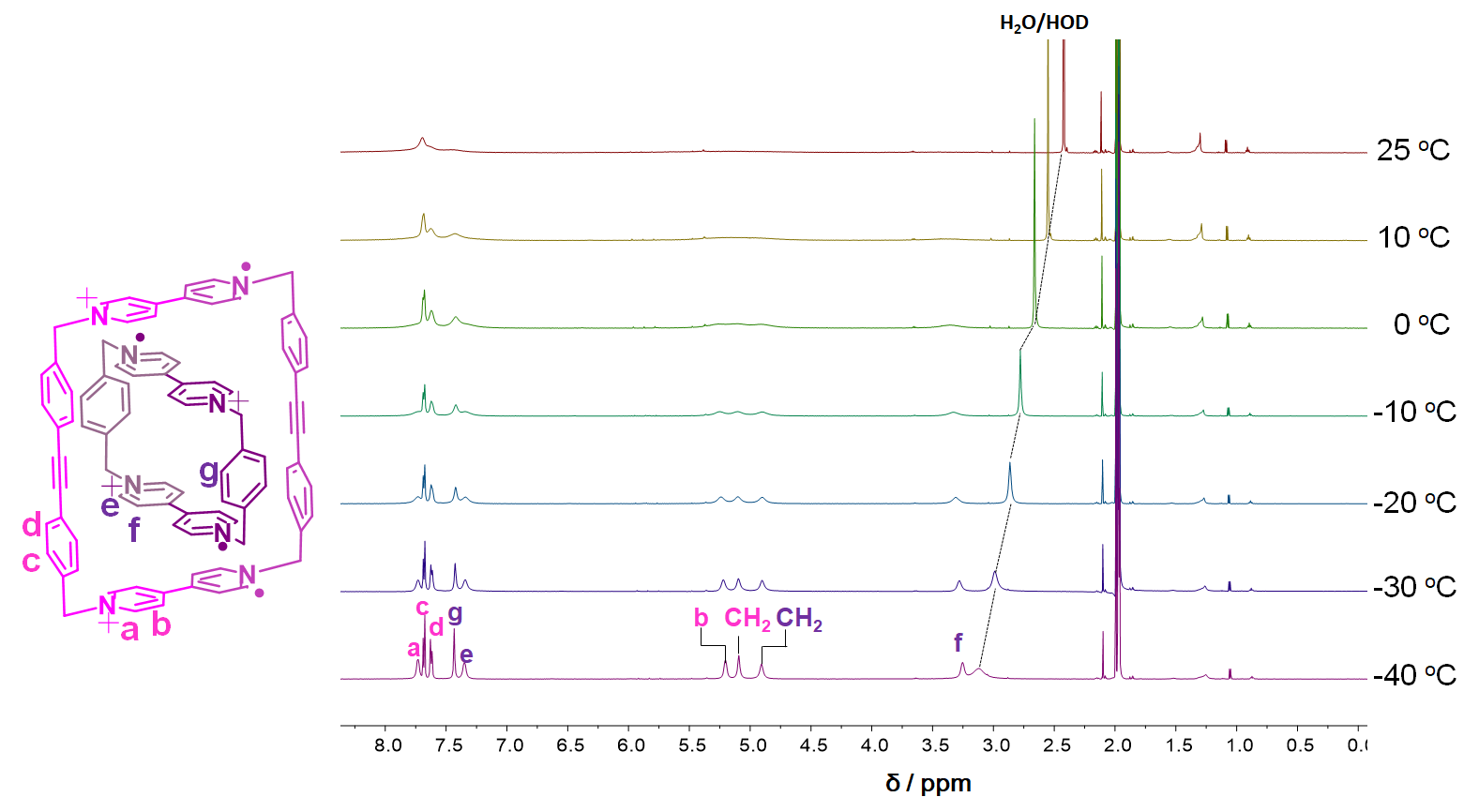


**Supplementary Figure 5.** ^1^H NMR Spectra on a mixture of **CBPQT^2(•+)^** and **1^2(•+)^** (1.0 mM each) recorded from −40 to 25 °C in CD_3_CN (600 MHz)


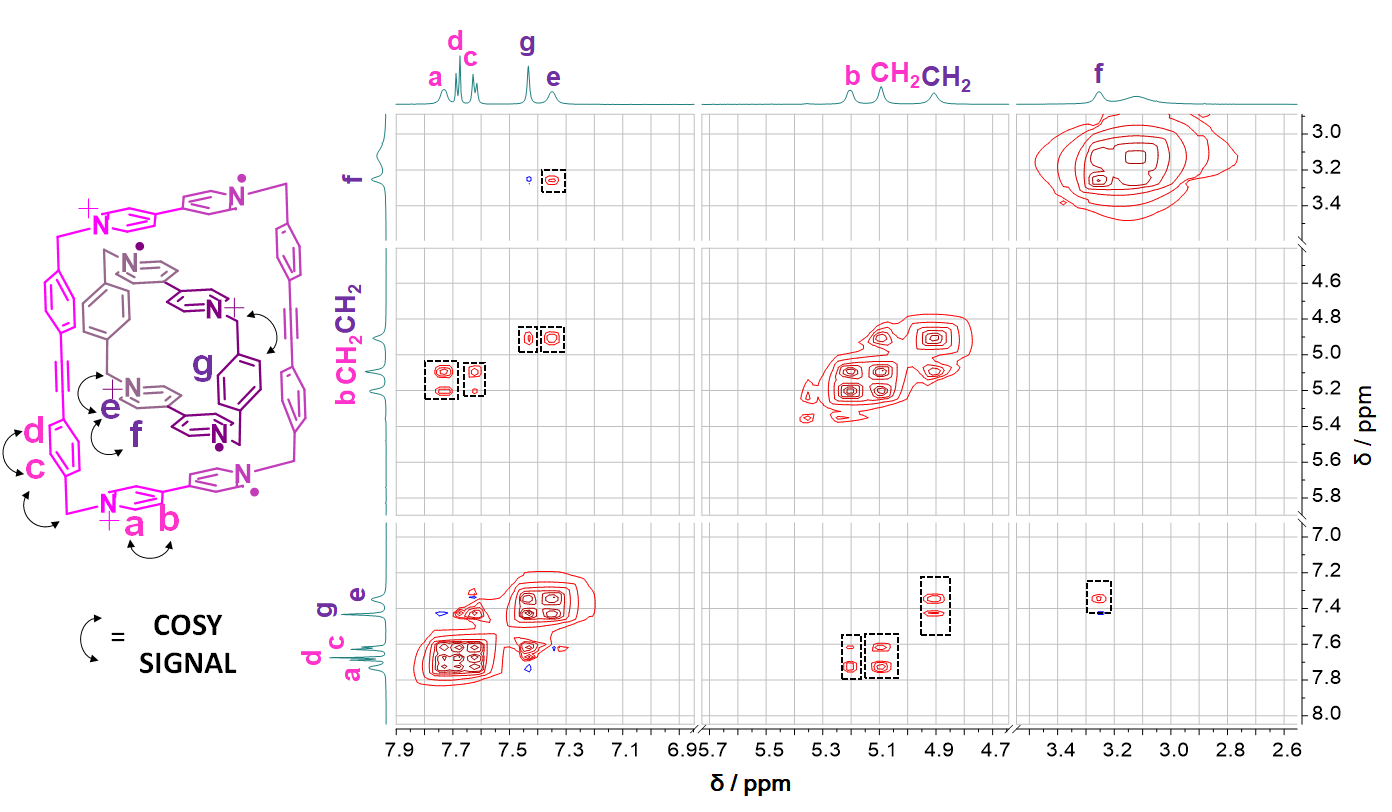


**Supplementary Figure 6.** ^1^H-^1^H COSY NMR Spectrum on a mixture of **CBPQT^2(•+)^** and **1^2(•+)^** (1.0 mM each) at −40 ^o^C (600 MHz, CD_3_CN)


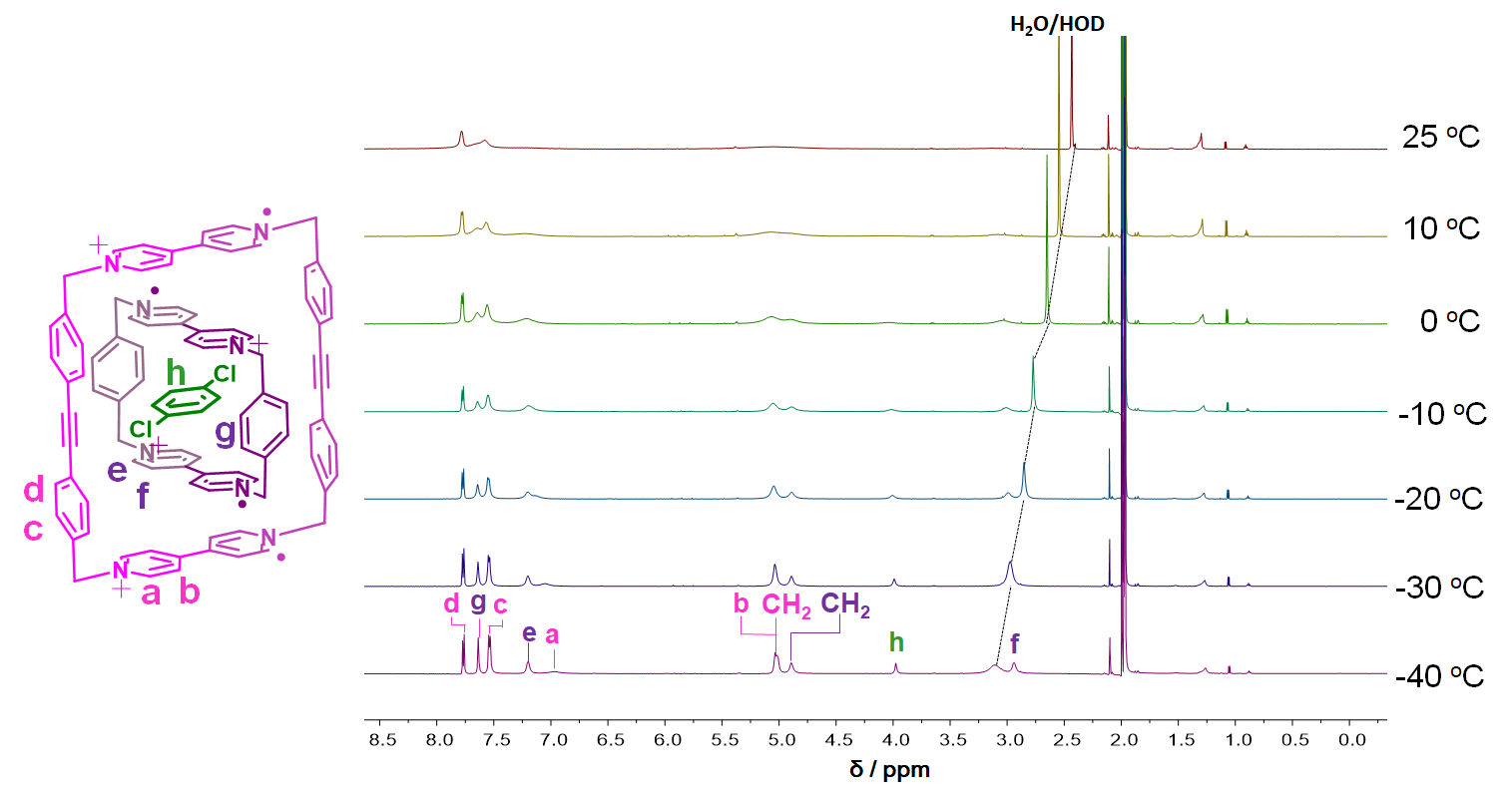


**Supplementary Figure 7.** ^1^H NMR Spectra recorded from −40 to 25 °C on a mixture of **CBPQT^2(•+)^**, **1^2(•+)^** and 1,4-dichlorobenzene (1.0 mM each) in CD_3_CN (600 MHz)


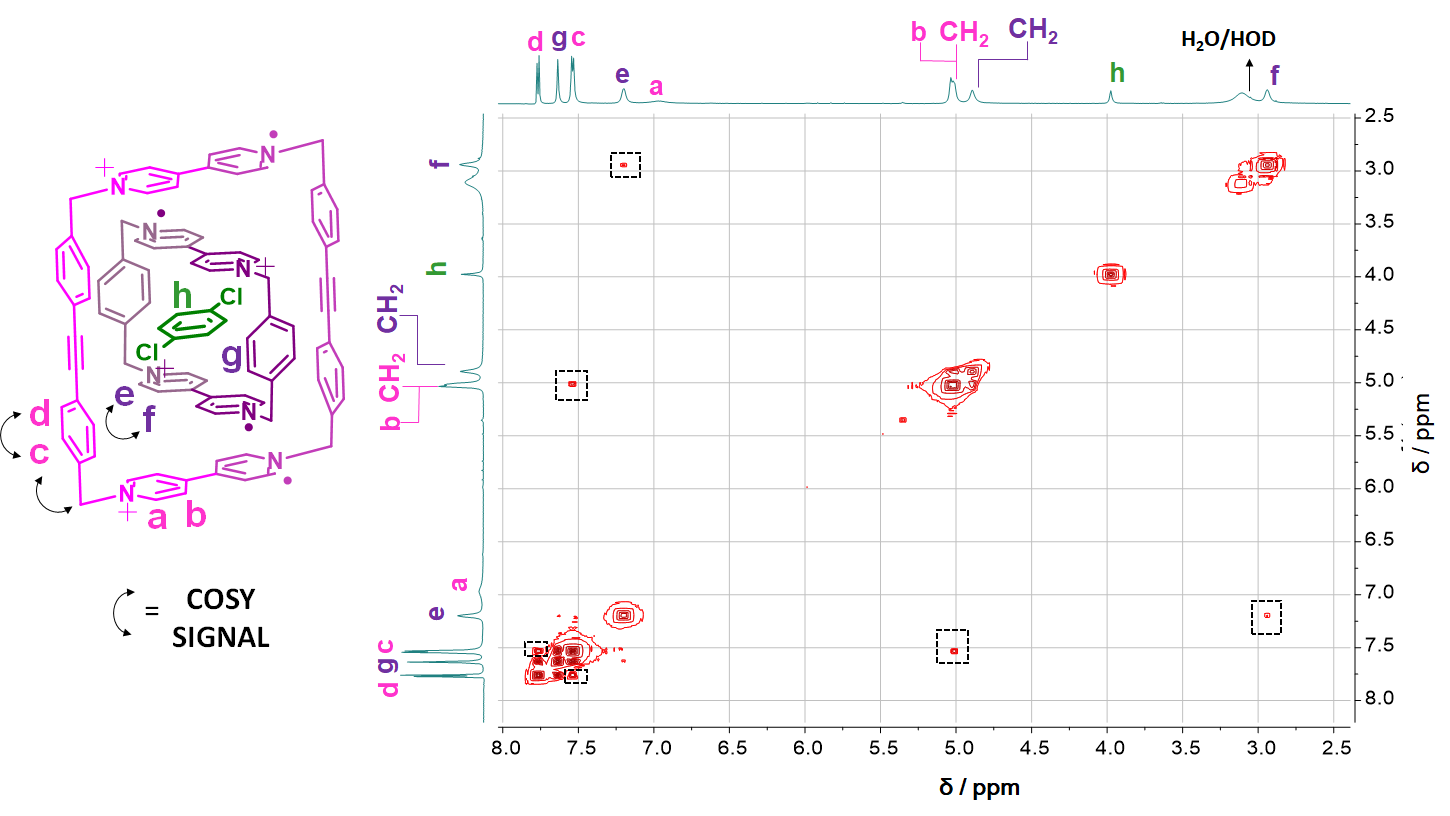


**Supplementary Figure 8.** ^1^H-^1^H COSY NMR Spectrum on a mixture of **CBPQT^2(•+)^**, **1^2(•+)^** and 1,4-dichlorobenzene (1.0 mM each) at −40 ^o^C (600 MHz, CD_3_CN).

**UV-Vis-NIR Titrations.** Stock solutions of the fully oxidized viologen derivatives **CBPQT•**4PF_6_, **1•**4PF_6_ were prepared in an N_2_ glovebox. The stock solutions were reduced over actviated Zn dust for 10‒15 min with stirring and then filtered to provide deep blue solutions of **CBPQT^2(+•)^** or **1^2(+•)^**. Syringes were employed to measure and dilute the radical stock solutions to the desired concentrations prior to measurements. Spectra were recorded from 1300 nm to 400 nm in a sealed 2 mm path length cells during titrating **CBPQT^2(+•)^** (0 to 10 equiv) into **1^2(+•)^**. Binding constant was obtained by fitting fit a 1:1 isotherm according to literature ^2^.


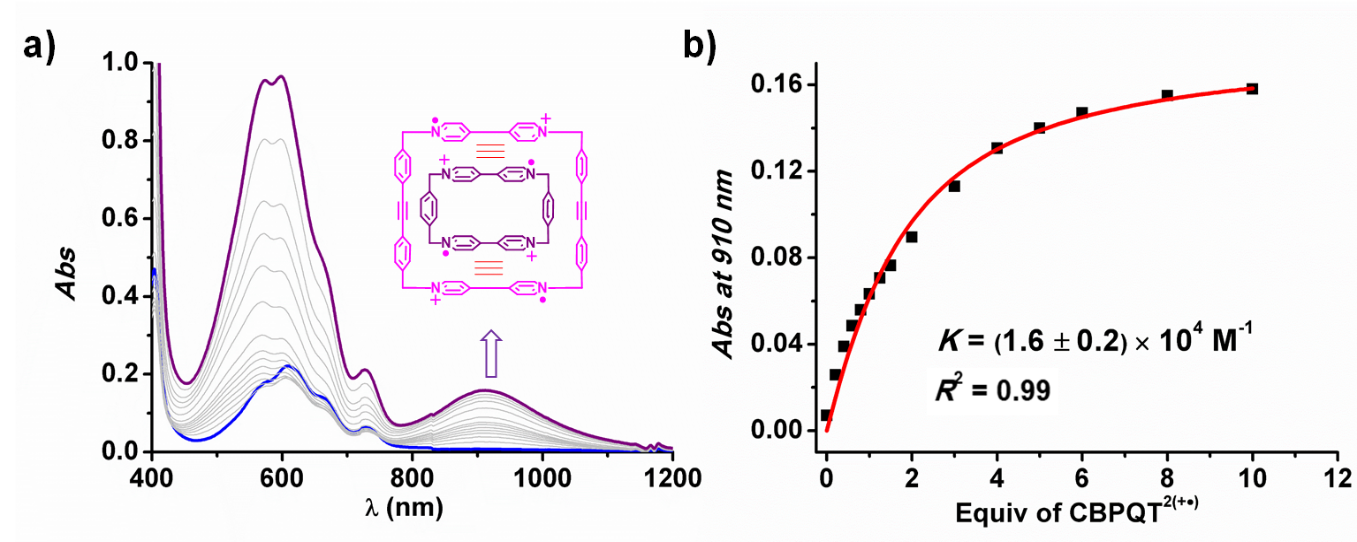


**Supplementary Figure 9.** a) Stacked UV-Vis-NIR spectra obtained by titrating **CBPQT^2(+•)^** (0 to 10 equiv) into a solution of **1^2(+•)^** (0.050 mM, MeCN, optical length: 2mm); b) Binding isotherm simulation.


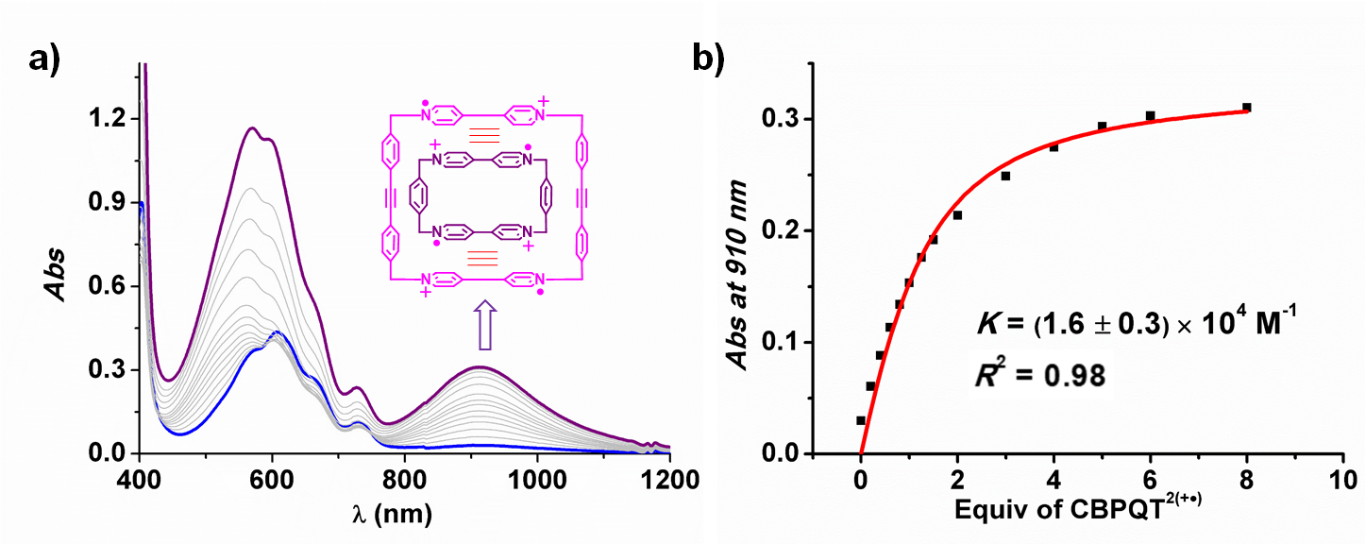


**Supplementary Figure 10.** a) Stacked UV-Vis-NIR spectra obtained by titrating **CBPQT^2(+•)^** (0 to 8 equiv) into a solution of **1^2(+•)^** (0.10 mM); b) Binding isotherm simulation. Solvent: MeCN. Optical length: 2mm.


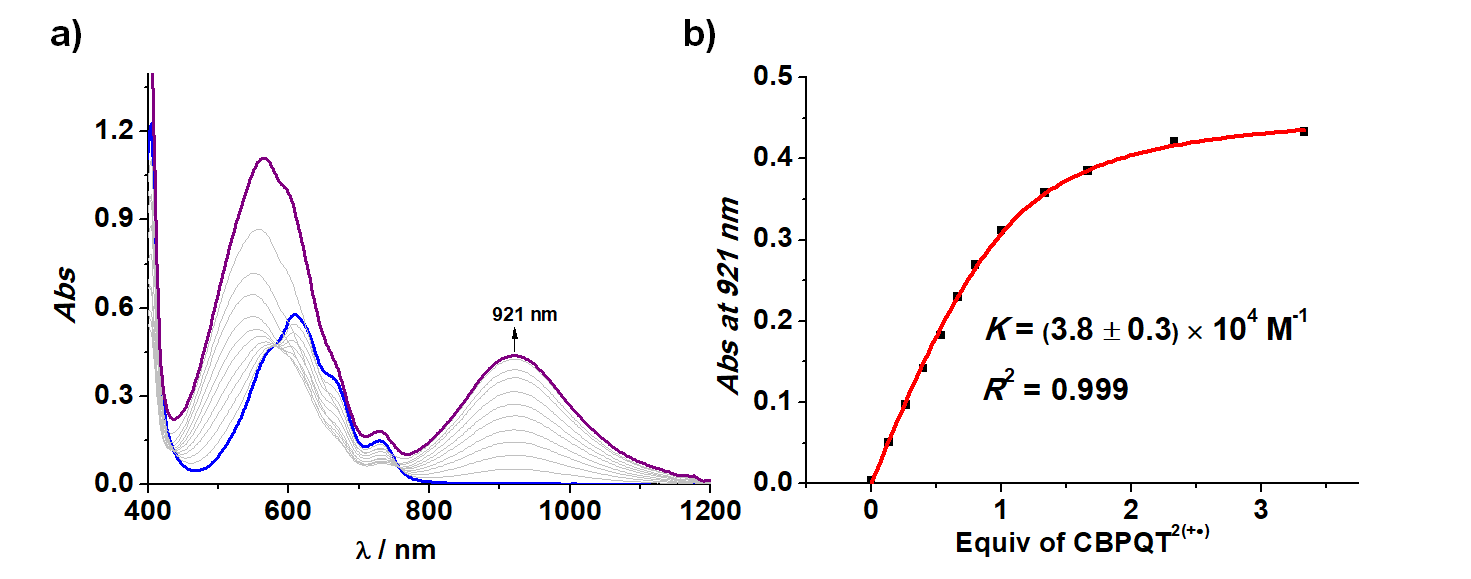


**Supplementary Figure 11.** a) Stacked UV-Vis-NIR spectra obtained by titrating **CBPQT^2(+•)^** (0 to 8 equiv) into a mixture solution of **1^2(+•)^** (0.15 mM) and 1,4-dichlorobenzene (0.30 mM); b) Binding isotherm simulation. Solvent: MeCN. Optical length: 2mm.


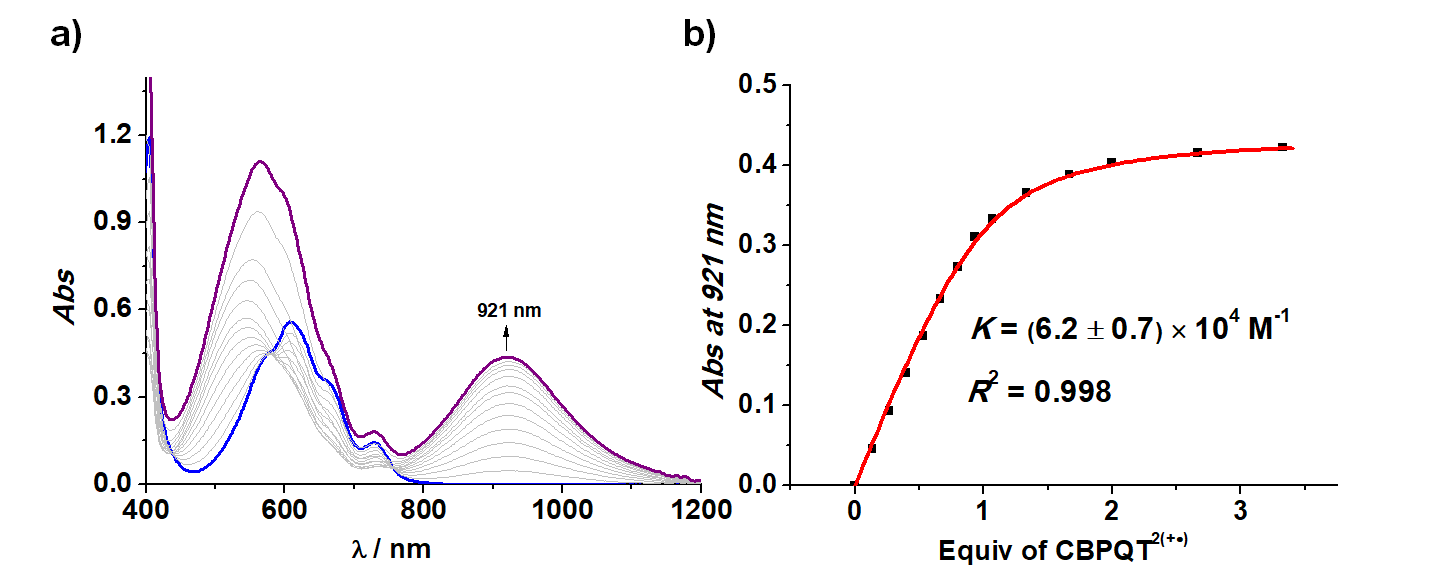


**Supplementary Figure 12.** a) Stacked UV-Vis-NIR spectra obtained by titrating **CBPQT^2(+•)^** (0 to 8 equiv) into a mixture solution of **1^2(+•)^** (0.15 mM) and 1,4-dichlorobenzene (1.0 mM); b) Binding isotherm simulation. Solvent: MeCN. Optical length: 2mm.


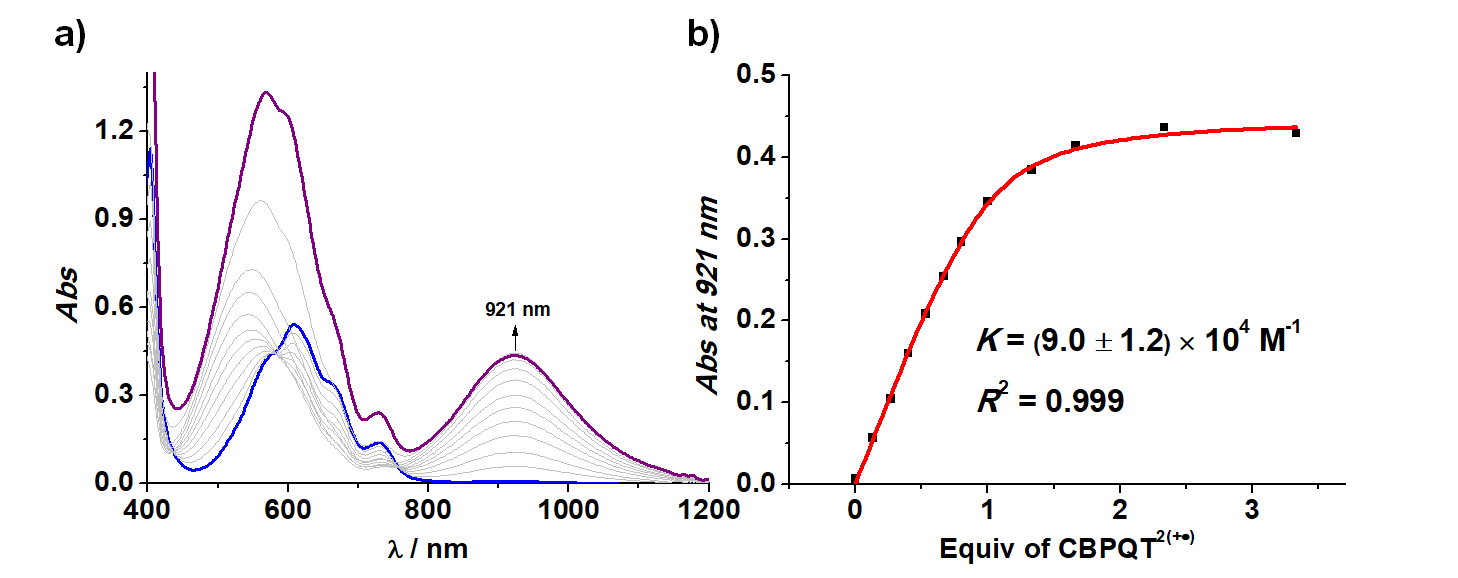


**Supplementary Figure 13.** a) Stacked UV-Vis-NIR spectra obtained by titrating **CBPQT^2(+•)^** (0 to 8 equiv) into a mixture solution of **1^2(+•)^** (0.15 mM) and 1,4-dichlorobenzene (3.0 mM); b) Binding isotherm simulation. Solvent: MeCN. Optical length: 2mm.

**Cyclic Voltammograms.** Samples for cyclic voltammetry were prepared using an electrolyte solution of 0.1 M [Bu_4_N][PF_6_] in MeCN that was sparged with Ar to remove O_2_. The cyclic voltammograms presented in the main text of the manuscript were recorded under Ar or N_2_ using a glassy carbon working electrode, a Pt wire or Pt mesh counter electrode, a silver wire quasi-reference electrode, and an internal standard of ferrocene. Scan rate viable CV data is presented here that was not included in the main text.

When the scan rate increased from 0.2 V/s to 50 V/s, the first reduction peak of the complex solution splits into two small overlapping peaks at more positive potentials. A mechanism was proposed to explain this phenomenon: since the two viologen pimers of [**CBPQT**⊂**1**]^4(+•)^ are distant from each other, they can be regarded as individual viologen radicals without interactions between each other in CVs. When the first electron in the viologen pimer is removed, a bisradical hexacationic complex [**CBPQT**⊂**1**]^4+2(+•)^ is generated and undergoes fast dissociation on account of significantly weakened association. At lower slow scan-rate, the bisradical complex [**CBPQT**⊂**1**]^4+2(+•)^ disassembles in solution on a time scale which is fast compared with the sweep in voltage, resulting in both **1^2(+•)^** and **CBPQT^2(+•)^**  being oxidized as separate compounds, giving rise to only one peak. At high scan rates, however, the rate is faster than the disassembly of the bisradical complex [**CBPQT**⊂**1**]^4+2(+•)^. It has a less favorable oxidation potential compared with that of [**1**⊂**CBPQT**]^4(+•)^ on account of the increase in the positive charge which renders this second oxidation potential more positive than the first one. Similar trends were also observed for the [**CBPQT**⊂**BIPY**]^3(+•)^ complex.


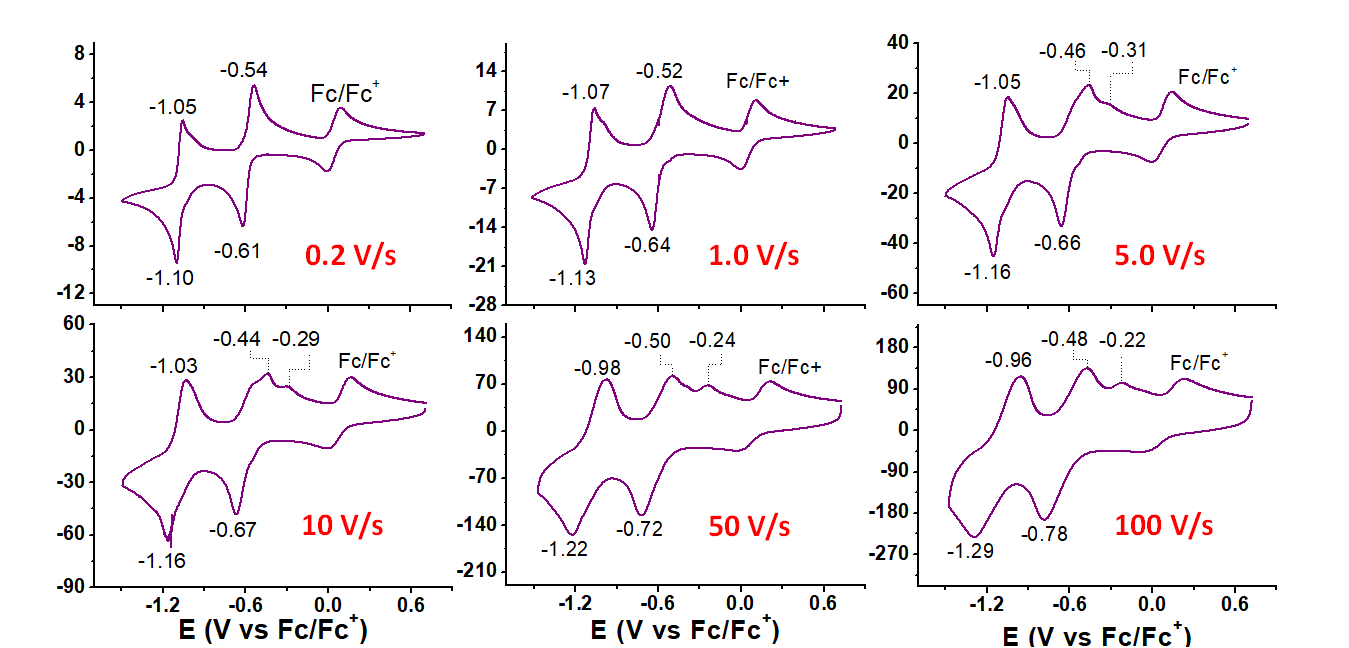


**Supplementary Figure 14.** Cyclic voltammograms of a 1:1 molar ratio mixture of **1^4+^** and **CBPQT^4+^** (0.20 mM each) in 0.1 M Bu_4_NPF_6_ in MeCN with potentials referenced to an internal standard of ferrocene (0.20 mM).

**Crystallographic Characterization**

**1•4PF_6_**

*a) Crystal Data.* Triclinic, space group $P\bar{1}$ (*a* = 19.2326(12), *b* = 19.9012(12), *c* = 21.5267(13) Å, *α* = 65.728(3), β = 69.007(3), γ = 70.594(4)°, *V*= 6844.9(8) Å^3^, *Z* = 6, *T* = 99.99 K, μ(CuKα) = 1.994 mm^-1^, *D_calc_* = 1.406 g/mm^3^, 23167 reflections measured (4.66 ≤ 2Θ ≤ 133.692), 23167 unique (*R*_sigma_ = 0.046) which were used in all calculations. The final *R*_1_ was 0.090 (*I* > 2*σ*(*I*)) and *wR*_2_ was 0.264 (all data).

*b) Refinement Details.* The structure was initially refined using the hklf4 file with the XL (Sheldrick, 2008) refinement package employing Least Squares minimization. Further refinement was performed against the hklf5 file. Hydrogen atom positions were placed using a riding model. The asymmetric unit contained several molecules of MeCN, and when possible, the atomic coordinates of these solvent molecules were freely refined. Disorder of some of the solvent molecules, as well as two of the PF_6_^‒^ anions, was modeled and refined, though some of the resulting atomic positions could only be refined isotropically. Lastly, the bypass procedure in Platon^3^ was applied to the hklf5 file to remove electron density resulting from additional solvent molecules that were too disordered to allow for the location of atomic positions. Final refinement was performed against the hklf5 file that had been processed in this manner.

**
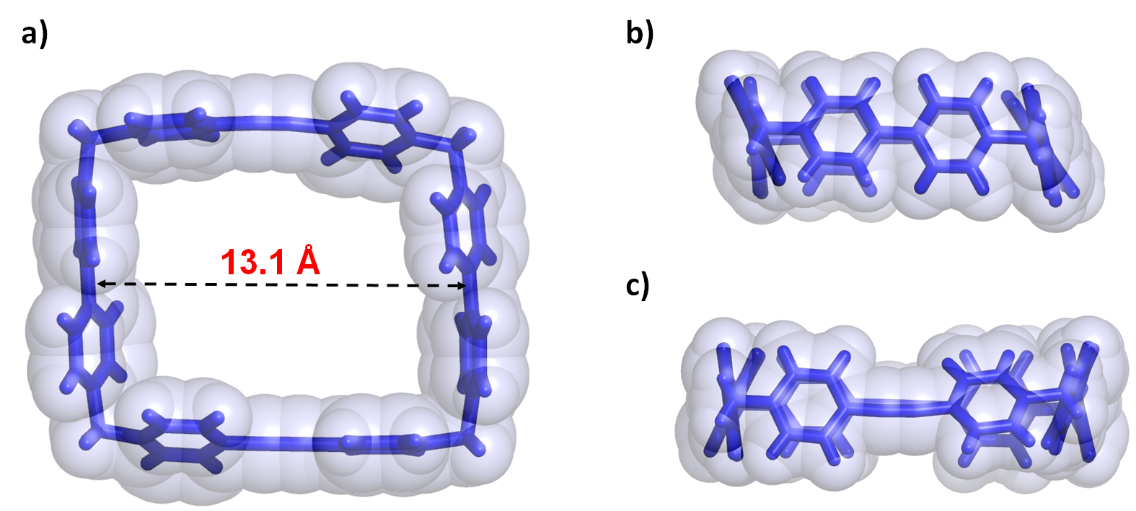
**

**Supplementary Figure 15.** Crystal structure of **1^4+^** depicted as tubular representation with transparent space-filling surfaces. a) View of **1^4+^** in the planes of four N atoms parallel to the page; b) View of **1^4+^** with the diphenylenethyne fragment aligned lengthwise perpendicular to the plane; c) View of **1^4+^** with the viologen units aligned lengthwise perpendicular to the plane. Counterions and solvent molecules are omitted for the sake of clarity.

**[CBPQT⊂1] •4PF_6_**

*a) Crystal Data.* Orthorhombic, space group *Cccm*, *a* = 25.154(3), *b* = 28.375(3), *c* = 16.7256(13) Å, *α* = *β* = *γ* = 90°, *V*= 11937.8(19) Å^3^, *Z* = 8, *T* = 100.03 K, *μ*(CuKα) = 0.912 mm^-1^, *D_calc_* = 1.170 g/mm^3^, 14077 reflections measured (4694 ≤ 2Θ ≤ 118.496), 4401 unique (*R*_int_ = 0.099, *R*_sigma_ = 0.074) which were used in all calculations. The final *R*_1_ was 0.101 (*I* > 2*σ*(*I*)) and *wR*_2_ was 0.337 (all data)

*b) Refinement Details.* The asymmetric unit contains several molecules of MeCN, and when possible, the atomic coordinates of these solvent molecules were freely refined. Some atomic positions could not be fully refined with anisotropic displacement parameters on account of disorder and/or insufficient strength of diffraction data, and as a consequence, some of the atomic positions of these solvent molecules were refined isotropically. One of these latter MeCN molecules was arranged within the asymmetric unit such that the symmetry elements of the space group provide infinite columns of closely spaced carbon and nitrogen atoms within the extended lattice. This chemically impossible result can be explained by disorder of aligned MeCN molecules within narrow channels in the superstructure. The solvent masking routine in Olex2 was used to account for electron density resulting from additional solvent molecules that were too disordered to allow for the location or refinement of atomic positions. This latter processing accounts for apparent voids within the cavity of the smaller cyclophane and within infinite narrow channels that are similar to, but crystallographically distinct from, those in which disordered columns of MeCN were identified.

**
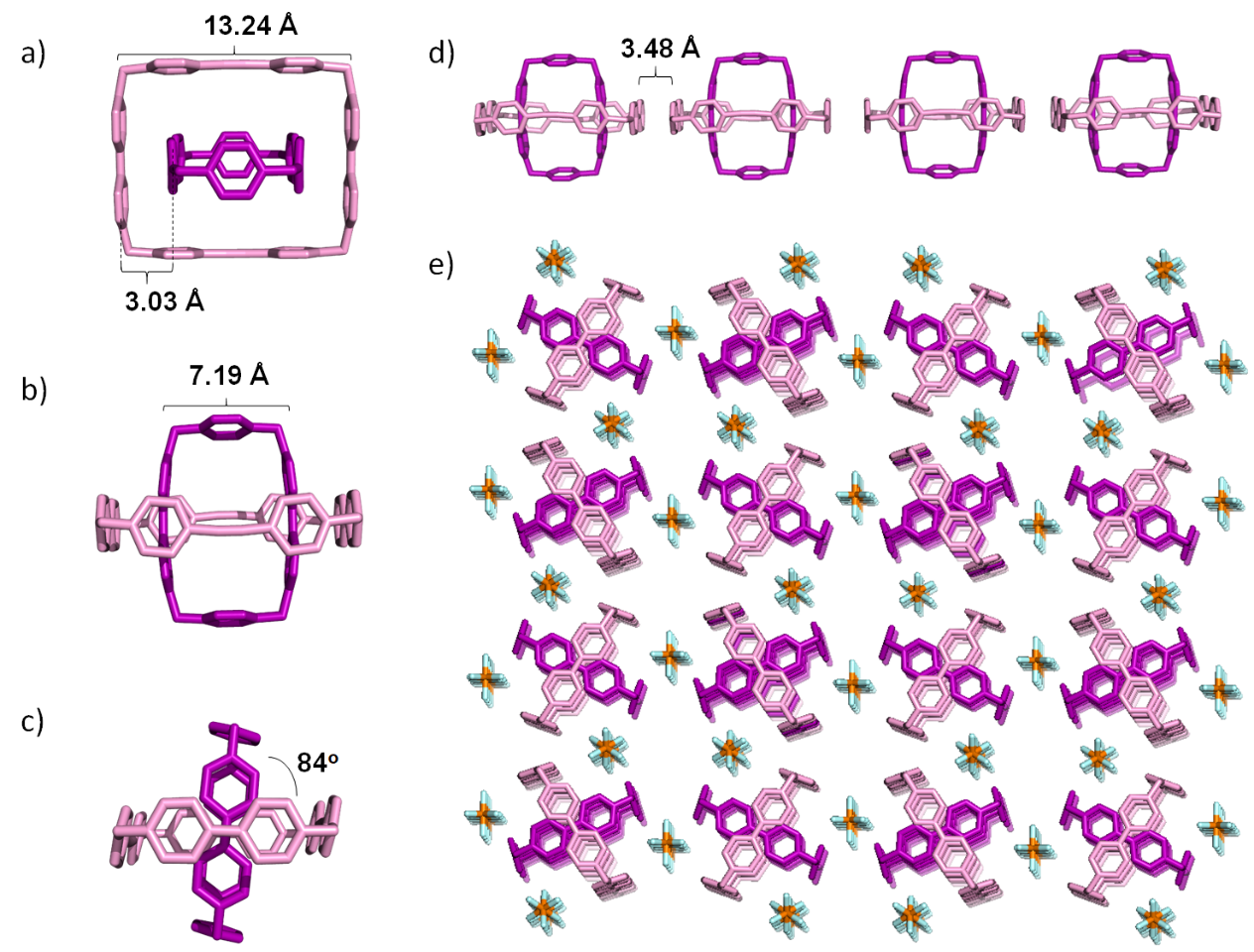
**

**Supplementary Figure 16.** Solid-state (super)structures of **[CBPQT⊂1]•**4PF**_6_**: (a) View of **[CBPQT⊂1]^4(+•)^** with the viologen units of **CBPQT^2(+•)^** aligned lengthwise perpendicular to the page; (b) View of **[CBPQT⊂1]^4(+•)^** with the viologen units of **1^2(+•)^** aligned lengthwise perpendicular to the page; (c) View of **[CBPQT⊂1]^4(+•)^** with the planes of the viologen units parallel to the page. (d) Side on view of 1D column of **[CBPQT⊂1]^4(+•)^** which shows the intermolecular packing between viologen units of **1^2(+•)^**; (e) View down the [−1, −1, 1] vector of the solid-state superstructure of **[CBPQT⊂1]•**4PF_6_. Counterions molecules are omitted in a)-d) for the sake of clarity.

**[1⊂CBPQT⊂DCB]•4PF_6_**

*a) Crystal Data.* Orthorhombic, space group *Pccn* (no. 56), *a* = 24.9565(7), *b* = 27.8623(9), *c* = 16.5996(4) Å, *V*= 11542.5(6) Å^3^, *Z* = 4, *T* = 100.06 K, *μ*(CuKα) = 1.776 mm^-1^, *D_calc_* = 1.227 g/mm^3^, 45474 reflections measured (4.754 ≤ 2Θ ≤ 133.35), 10068 unique (*R*_int_ = 0.035, *R*_sigma_ = 0.035) which were used in all calculations. The final *R*_1_ was 0.061 (*I* > 2*σ*(*I*)) and *wR*_2_ was 0.188 (all data).

*b) Refinement Details.* Two reflections were omitted because they were behind the beam stop of the diffractometer.

**
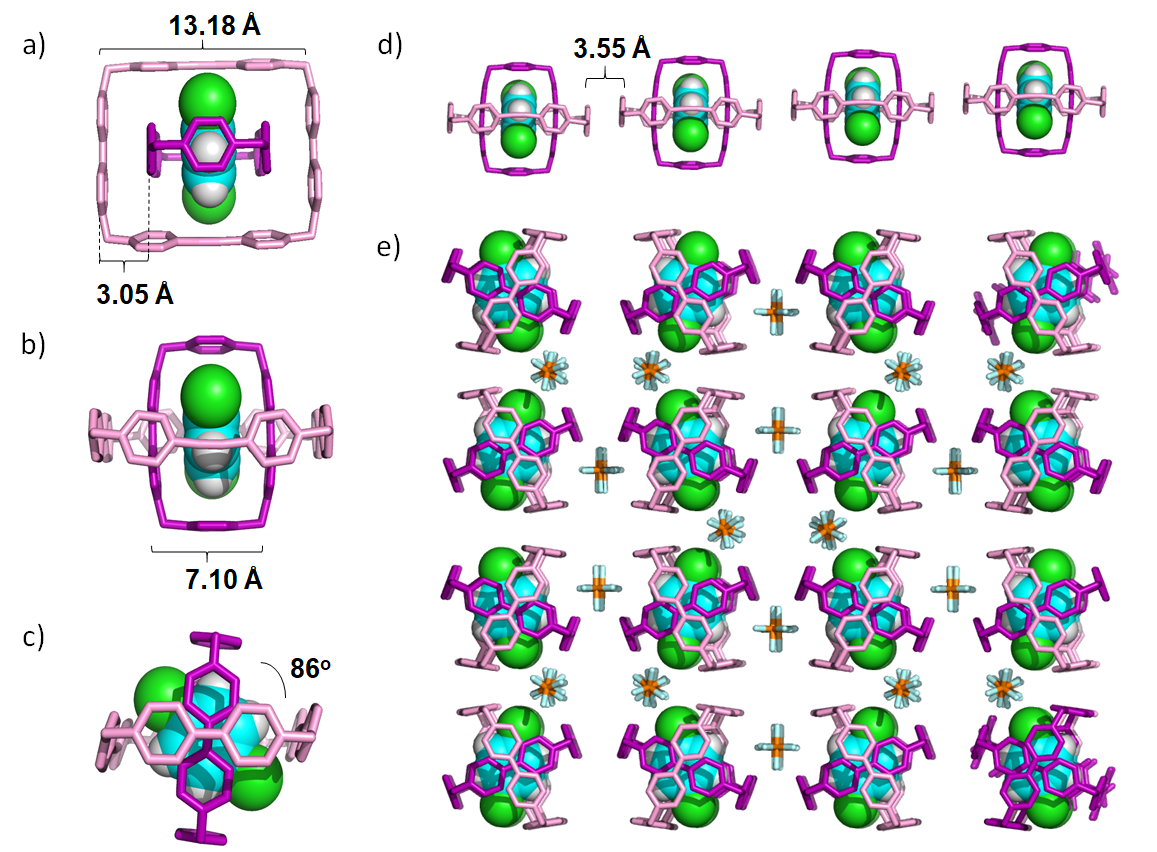
**

**Supplementary Figure 17.** Solid-state (super)structures of **[DCB ⊂CBPQT⊂1]•4PF_6_**: (a) View of **[DCB ⊂CBPQT⊂1]^4(+•)^** with the viologen units of **CBPQT^2(+•)^** aligned lengthwise perpendicular to the page; (b) View of **[1⊂CBPQT⊂DCB]^4(+•)^** with the viologen units of **1^2(+•)^** aligned lengthwise perpendicular to the page; (c) View of **[DCB ⊂CBPQT⊂1]^4(+•)^** with the planes of the viologen units parallel to the page. (d) Side on view of 1D column of **[DCB ⊂CBPQT⊂1]^4(+•)^** that shows the intermolecular packing between viologen units of **1^2(+•)^**; (e) View down the [−1, −1, 1] vector of the solid-state superstructure of **[DCB ⊂CBPQT⊂1]•**4PF_6_. **DCB**: 1,4-Dichlorobenzene.

**[DBB ⊂CBPQT⊂1] •4PF_6_:**

*a) Crystal Data.* Orthorhombic, space group *Cccm* (no. 66), *a* = 22.9893(11), *b* = 27.1658(13), *c* = 33.4104(16), *V*= 20865.6(17) Å^3^, *Z* = 8, *T* = 100.0 K, μ(CuKα) = 2.351 mm^-1^, *D_calc_* = 1.336 g/mm^3^, 30423 reflections measured (5.29 ≤ 2Θ ≤ 130.35), 8638 unique

**
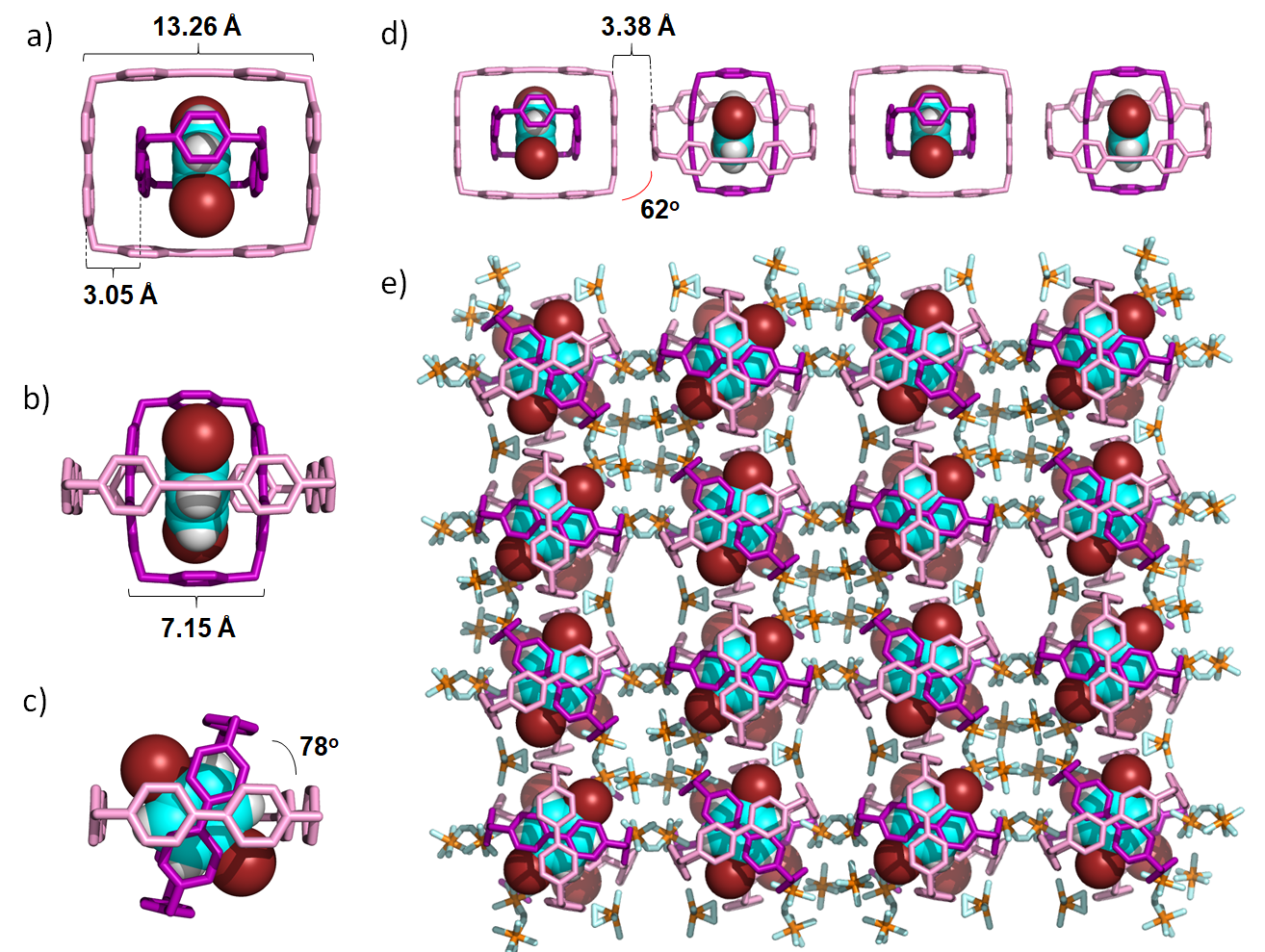
**

**Supplementary Figure 18.** Solid-state (super)structures of **[DBB ⊂CBPQT⊂1 ]•**4PF_6_: (a) View of **[DBB ⊂CBPQT⊂1]^4(+•)^** with the viologen units of **CBPQT^2(+•)^** aligned lengthwise perpendicular to the page; (b) View of **[DBB ⊂CBPQT⊂1]^4(+•)^** with the viologen units of **1^2(+•)^** aligned lengthwise perpendicular to the page; (c) View of **[DBB ⊂CBPQT⊂1]^4(+•)^** with the planes of the viologen units parallel to the page. (d) Side on view of 1D column of **[DBB ⊂CBPQT⊂1]^4(+•)^** that shows the intermolecular packing between viologen units of **1^2(+•)^**; (e) View down the [−1, −1, 1] vector of the solid-state superstructure of **[DBB ⊂CBPQT⊂1]•**4PF_6_. **DBB**: 1,4-Dibromobenzene.

(*R*_int_ = 0.079, *R*_sigma_ = 0.086) which were used in all calculations. The final *R*_1_ was 0.133 (*I* > 2*σ*(*I*)) and *wR*_2_ was 0.365 (all data).

*b) Refinement Details.* Distance restraints were imposed on the disordered PF_6_^‒^ anions. Rigid bond restraints were imposed on the displacement parameters as well as restraints on similar amplitudes separated by less than 1.7 Å. on the disordered PF_6_^‒^ anions. Distance restraints and ISOR was imposed on the MeCN solvent molecules.

**[DTP ⊂CBPQT⊂1] •4PF_6_:**

*a) Crystal Data.* Triclinic, space group $P\bar{1}$ (no. 2), *a* = 13.1693(7), *b* = 13.2133(7), *c* = 16.9227(7), *α* = 101.344(3), *β* = 92.167(3), *γ* = 118.552(3)°, *V*= 2506.2(2) Å^3^, *Z* = 1, *T* = 100.15 K, μ(CuKα) = 1.610 mm^-1^, *D_calc_* = 1.443 g/mm^3^, 7889 reflections measured (7.734 ≤ 2Θ ≤ 131.838), 7889 unique (*R*_int_ = 0.071, *R*_sigma_ = 0.073) which were used in all calculations. The final *R*_1_ was 0.149 (*I* > 2*σ*(*I*)) and *wR*_2_ was 0.355 (all data).

*b) Refinement Details.* Rigid bond restraints were imposed globally on the displacement parameters as well as restraints on similar amplitudes separated by less than 1.7 Å. ISOR restraint was imposed on an MeCN solvent molecule. The crystal under investigation was found to be non-merohedrally twinned. The orientation matrices for the two components were identified using the program Cell Now (Sheldrick, 2005), and the data were processed using both orientation matrices with SAINT, The exact twin matrix identified by the integration program was found to be (-0.00283, 0.99523, -0.00038 / 1.00471, 0.00284,

0.00027 / -0.57695, -0.57654, -1.00001). The second domain is rotated from first domain by 169.8% about the real lattice *b* axis. The absorption correction was carried out using TWINABS V2008/4 to create an hklf5 file which was used in all refinements; the structure was solved using direct methods with only the non-overlapping reflections of component 1. The twin fraction refined to a value of 0.436(4).

**
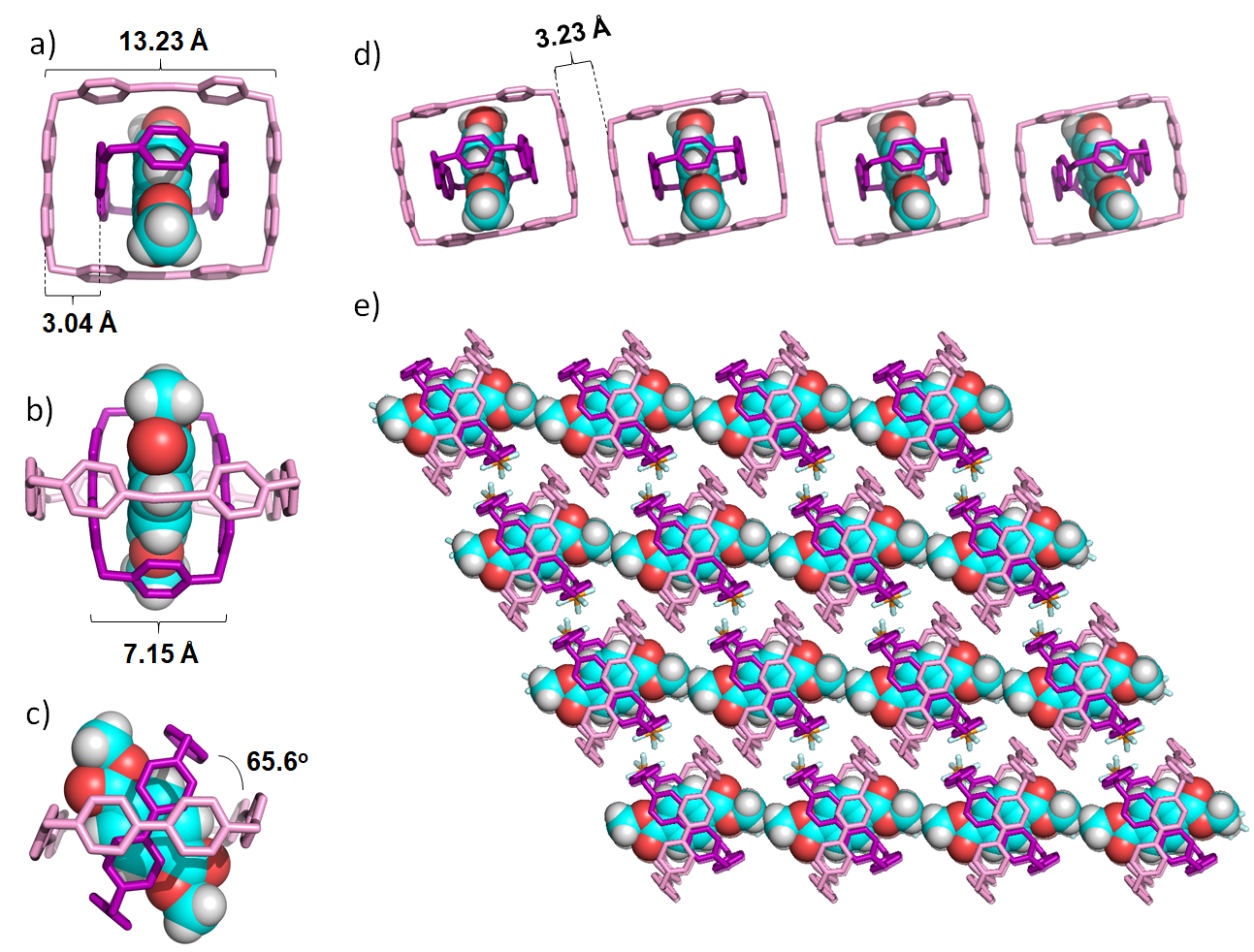
**

**Supplementary Figure 19.** Solid-state (super)structures of **[DTP ⊂CBPQT⊂1]•**4PF_6_: (a) View of **[1⊂CBPQT⊂DTP]^4(+•)^** with the viologen units of **CBPQT^2(+•)^** aligned lengthwise perpendicular to the page; (b) View of **[DTP ⊂CBPQT⊂1]^4(+•)^** with the viologen units of **1^2(+•)^** aligned lengthwise perpendicular to the page; (c) View of **[DTP ⊂CBPQT⊂1]^4(+•)^** with the planes of the viologen units parallel to the page. (d) Side on view of 1D column of **[DTP ⊂CBPQT⊂1 ]^4(+•)^** that shows the intermolecular packing between viologen units of **1^2(+•)^**; (e) View down the [−1, −1, 1] vector of the solid-state superstructure of **[ DTP ⊂CBPQT⊂1]•**4PF_6_. **DTP**: 1,4-Dimethylterephthalate.

**[BPTP ⊂CBPQT⊂1] •**4PF_6_**:**

*a) Crystal Data.* Triclinic, space group $P\bar{1}$ (no. 2), *a* = 13.257(3), *b* = 13.407(2), *c* = 16.845(4), *α* = 75.445(11), *β* = 88.351(13), *γ* = 60.638(10)°, *V*= 2509.8(9) Å^3^, *Z* = 1, *T* = 100.04 K, μ(CuKα) = 1.622 mm^-1^, *D_calc_* = 1.475 g/mm^3^, 22866 reflections measured (7.7 ≤ 2Θ ≤ 130.174), 8416 unique (*R*_int_ = 0.036, *R*_sigma_ = 0.042) which were used in all calculations. The final *R*_1_ was 0.048 (*I* > 2*σ*(*I*)) and *wR*_2_ was 0.128 (all data).

*b) Refinement Details.* 2 reflections were omitted because they were behind the beam stop.

**
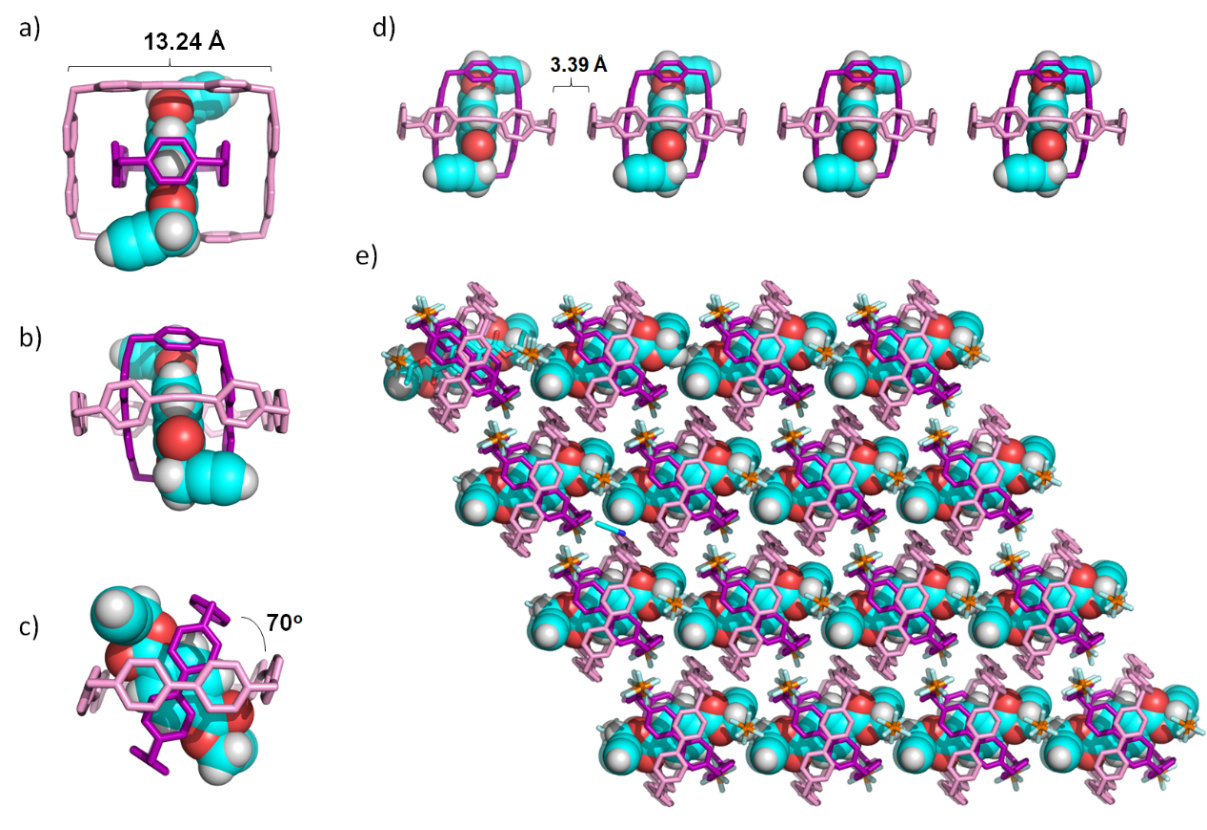
**

**Supplementary Figure 20.** Solid-state (super)structures of **[BPTP ⊂CBPQT⊂1]•**4PF_6_: (a) View of **[BPTP⊂CBPQT⊂1]^4(+•)^** with the viologen units of **CBPQT^2(+•)^** aligned lengthwise perpendicular to the page; (b) View of **[1⊂CBPQT⊂BPTP]^4(+•)^** with the viologen units of **1^2(+•)^** aligned lengthwise perpendicular to the page; (c) View of **[BPTP ⊂CBPQT⊂1]^4(+•)^** with the planes of the viologen units parallel to the page. (d) Side on view of 1D column of **[BPTP ⊂CBPQT⊂1]^4(+•)^** that shows the intermolecular packing between viologen units of **1^2(+•)^**; (e) View down the [−1, −1, 1] vector of the solid-state superstructure of **[BPTP ⊂CBPQT⊂1]•**4PF_6_. **BPTP**: Bis(propargyl)tere-phthalene.

**[BAB ⊂CBPQT⊂1] •4PF_6_:**

*a) Crystal Data.* Triclinic, space group $P\bar{1}$ (no. 2), *a* = 13.1492(8), *b* = 13.3934(9), *c* = 16.8873(11) Å, *α* = 90.192(4), *β* = 102.626(3), *γ* = 119.188(3)°, *V*= 2512.5(3) Å^3^, *Z* = 1, *T* = 99.99 K, *μ*(CuKα) = 1.589 mm^-1^, *D_calc_* = 1.438 g/mm^3^, 29337 reflections measured (7.624 ≤ 2Θ ≤ 130.6), 8421 unique (*R*_int_ = 0.034, *R*_sigma_ = 0.031) which were used in all calculations. The final *R*_1_ was 0.040 (*I* > 2σ(*I*)) and *wR*_2_ was 0.106 (all data).

*b) Refinement Details.* No special refinement necessary.

**
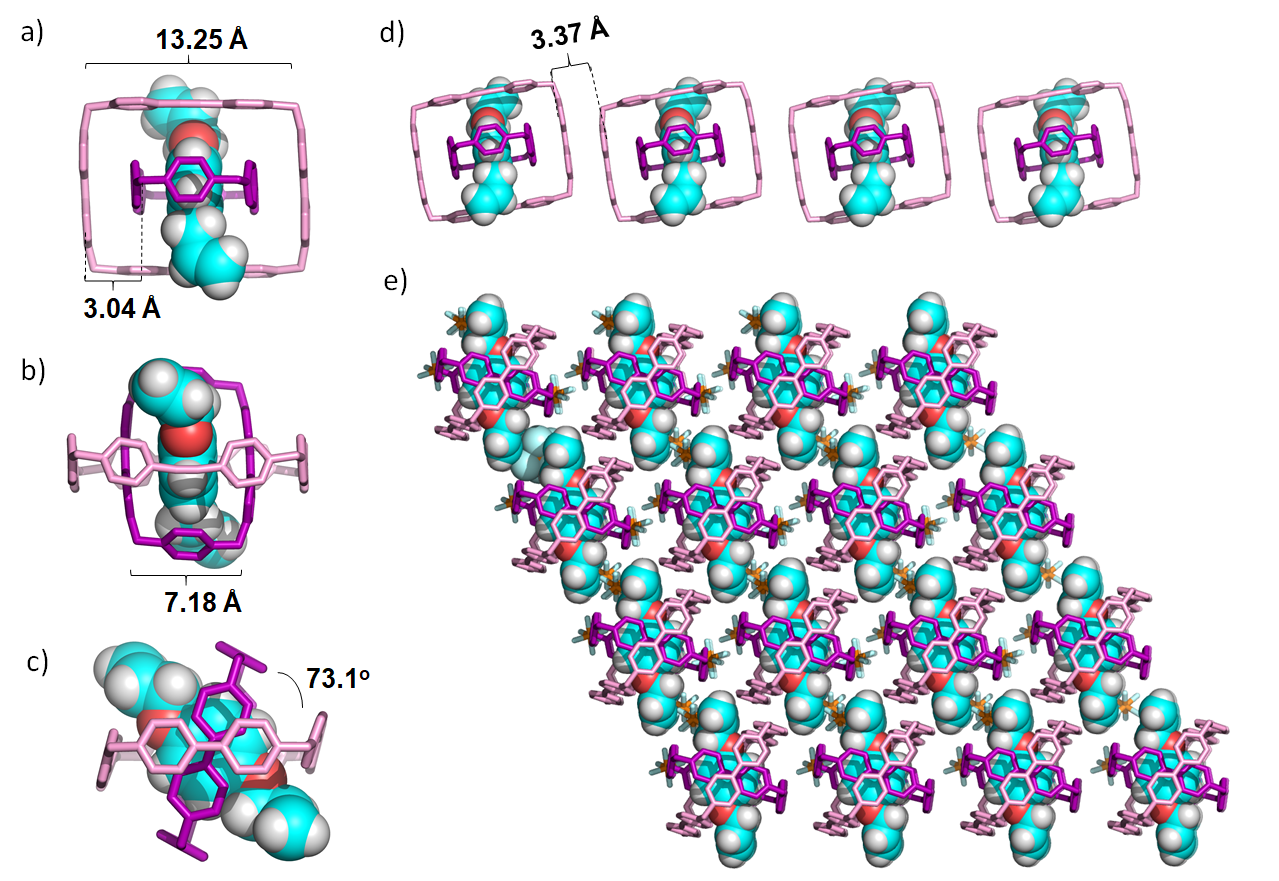
**

**Supplementary Figure 21.** Solid-state (super)structures of **[BAB ⊂CBPQT⊂1]•**4PF_6_: (a) View of **[BAB ⊂CBPQT⊂1]^4(+•)^** with the viologen units of **CBPQT^2(+•)^** aligned lengthwise perpendicular to the page; (b) View of **[BAB ⊂CBPQT⊂1]^4(+•)^** with the viologen units of **1^2(+•)^** aligned lengthwise perpendicular to the page; (c) View of **[BAB ⊂CBPQT⊂1]^4(+•)^** with the planes of the viologen units parallel to the page. (d) Side on view of 1D column of **[BAB ⊂CBPQT⊂1]^4(+•)^** that shows the intermolecular packing between viologen units of **1^2(+•)^**; (e) View down the [−1, −1, 1] vector of the solid-state superstructure of **[BAB ⊂CBPQT⊂1]•**4PF_6_. **BAB**: Bis(allyloxy)benzene.

**EPR Measurements.** Solutions of **CBPQT^2(+•)^** and **1^2(+•)^** were prepared in the same way as was described for preparing the samples utilized for UV-Vis-NIR measurements. After mixing and/or diluting the radical samples to the desired concentration, 100 μL of each sample was transferred to a quartz EPR tube (1.7 mm x 1.5 mm: outer x inner diameter) by syringe. The tubes were sealed with UV-cure resin under an N_2_ atmosphere.

The CW-EPR spectra of the samples were measured with a Bruker eleXsys-580 X-band (ca. 9.8 GHz) spectrometer equipped with a super-high-Q resonator (4122-SHQE). Typical parameters were: 100 kHz frequency modulation, 0.01 to 0.1 mT modulation amplitudes, 5 second time constant, and 20 second conversion time. Operating microwave powers in the range of 0.25 to 0.75 mW were confirmed to be linear with respect to signal intensity (no saturation of the spectra). For consistent comparison of EPR spectra between samples or of the same sample at different temperatures (Figure 3a), the tuning of the resonator and frequency of the spectrometer was kept constant (same Q value) between measurements.


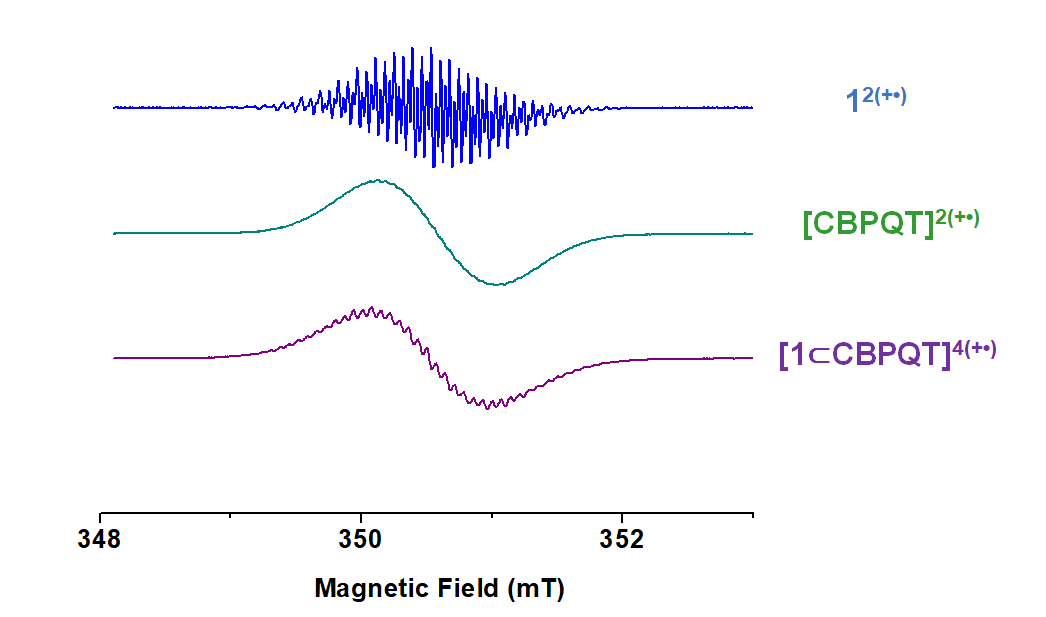


**Supplementary Figure 22.** EPR Spectra of solutions of **1^2(+•)^** (0.50 mM), **CBPQT^2(+•)^** (0.50 mM), **[CBPQT ⊂1 ]^4(+•)^** (0.25 mM) recorded in MeCN at room temperature.

**Supplementary Figure 23.** VT-EPR Spectra of crystalline sample (1mg) of **[CBPQT⊂1]•4PF_6_** from 293 K to 323 K.

**Supplementary Figure 24.** EPR spectrum of of **[CBPQT⊂1]•4PF_6_** (blue line) and **[*p*-C_6_H_4_Cl_2_⊂CBPQT⊂1]•4PF_6_** (red line) at 293 K in MeCN (0.50 mM for each).

**DFT Calculations**. DFT Calculations were performed using the M06-2XS^4^ functional with the 6-311G** basis set.^5^ The (super)structures were optimized including the D3 van der Waals correction in the presence of the Poisson-Boltzmann solvation model for MeCN (*ɛ* = 37.5 and *R_0_* = 2.18 Å) as implemented in Jaguar 8.2.^6-8^ Spin unrestricted calculations were applied to all molecules and complexes with unpaired electrons. The intermolecular interaction was probed further through examination of the molecular orbitals of the host-guest complex. The HOMO and HOMO-1 orbitals indicate, as expected, significant in-phase mixing between the SOMOs of the host and guest units. In contrast, the LUMO and LUMO-1 exhibit significant out-of-phase mixing of the orbitals on the guest with those on the host.

**^
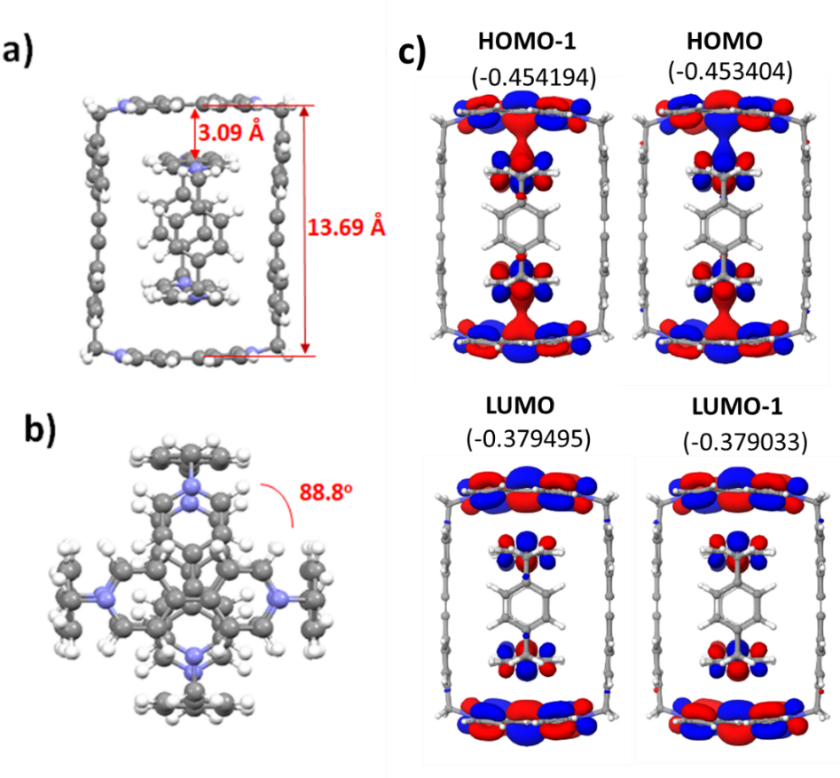
^**

**Supplementary Figure 25.** Optimized computational model (super)structures (a, b) and frontier molecular orbitals (c) of [**CBPQT** ⊂**1**]^4(+•)^ (orbital energies are provided in parentheses in units of Hartrees).

**Supplementary References**

(1) Barnes, J. C.; Juríček, M.; Vermeulen, N. A.; Dale, E. J.; Stoddart, J. F. *J. Org. Chem.* **2013**, *78*, 11962.

(2) Lipke, M. C.; Chang, T.; Wu, Y.; Arslan, H.; Xiao, H.; Wasielewski, M. R.; Goddard III, W. A.; Stoddart, J. F. *J. Am. Chem. Soc.* **2017**, *139*, 3986–3998.

(3)  Sluis, P. van der**;** Spek, A.L. *Acta Cryst.* **1990**, *46*, 194-201.

(4) [Houk](https://pubs.acs.org/author/Houk%2C+K+N), K. N.; [Menzer](https://pubs.acs.org/author/Menzer%2C+Stephan), S.; [Newton](https://pubs.acs.org/author/Newton%2C+Simon+P), S. P.;  [Raymo](https://pubs.acs.org/author/Raymo%2C+Fran%C3%A7isco+M), F. M.; [Stoddart](https://pubs.acs.org/author/Stoddart%2C+J+Fraser), J. F.; [Williams](https://pubs.acs.org/author/Williams%2C+David+J), D. J. J. Am. Chem. Soc. **1999**, *121*, 1479.

(5) (a) Zhao, Y.; Truhlar, D. Theor. *Chem. Acc.* **2008**, *120*, 215; (b) Zhao, Y.; Truhlar, D. G. *Acc. Chem. Res.* **2008**, *41*, 157.

(6) (a) Clark, T.; Chandrasekhar, J.; Spitznagel, G. W.; Schleyer, P. V. R. J. *Comput. Chem.* **1983**, *4*, 294; (b) Frisch, M. J.; Pople, J. A.; Binkley, J. S. *J. Chem. Phys.* **1984**, *80*, 3265; (c) Krishnan, R.; Binkley, J. S.; Seeger, R.; Pople, J. A. *J. Chem. Phys.* **1980**, *72*, 650; (d) McLean, A. D.; Chandler, G. S. *J. Chem. Phys*. **1980**, *72*, 5639.

(7) Tannor, D. J.; Marten, B.; Murphy, R.; Friesner, R. A.; Sitkoff, D.; Nicholls, A.; Honig, B.; Ringnalda, M.; Goddard, W. A., III *J. Am. Chem. Soc.* **1994**, *116*, 11875.

(8) Jaguar, version 8.2, Schrödinger, LLC, New York, NY, 2013
